# Eukaryotic Initiation Factor 5A2 localizes to actively translating ribosomes to promote cancer cell protrusions and invasive capacity

**DOI:** 10.1186/s12964-023-01076-6

**Published:** 2023-03-13

**Authors:** Arantxa Martínez-Férriz, Carolina Gandía, José Miguel Pardo-Sánchez, Alihamze Fathinajafabadi, Alejandro Ferrando, Rosa Farràs

**Affiliations:** 1 Centro de Investigación Príncipe Felipe, Valencia, Spain; 2 Instituto de Biología Molecular y Celular de Plantas, Consejo Superior de Investigaciones Científicas. Universidad Politécnica de Valencia, 46022 Valencia, Spain

**Keywords:** Eukaryotic translation initiation factor 5A2, TGFB1 signaling, Translating ribosomes, Cytoskeleton organization, Cell migration, Lung adenocarcinoma

## Abstract

**Background:**

Eukaryotic Initiation Factor 5A (eIF-5A), an essential translation factor, is post-translationally activated by the polyamine spermidine. Two human genes encode eIF-5A, being eIF5-A1 constitutively expressed whereas eIF5-A2 is frequently found overexpressed in human tumours. The contribution of both isoforms with regard to cellular proliferation and invasion in non-small cell lung cancer remains to be characterized.

**Methods:**

We have evaluated the use of eIF-5A2 gene as prognosis marker in lung adenocarcinoma (LUAD) patients and validated in immunocompromised mice. We have used cell migration and cell proliferation assays in LUAD lines after silencing each eIF-5A isoform to monitor their contribution to both phenotypes. Cytoskeleton alterations were analysed in the same cells by rhodamine-phalloidin staining and fluorescence microscopy. Polysome profiles were used to monitor the effect of eIF-5A2 overexpression on translation. Western blotting was used to study the levels of eIF-5A2 client proteins involved in migration upon TGFB1 stimulation. Finally, we have co-localized eIF-5A2 with puromycin to visualize the subcellular pattern of actively translating ribosomes.

**Results:**

We describe the differential functions of both eIF-5A isoforms, to show that eIF5-A2 properties on cell proliferation and migration are coincident with its features as a poor prognosis marker. Silencing of eIF-5A2 leads to more dramatic consequences of cellular proliferation and migration compared to eIF-5A1. Overexpression of eIF-5A2 leads to enhanced global translation. We also show that TGFβ signalling enhances the expression and activity of eIF-5A2 which promotes the translation of polyproline rich proteins involved in cytoskeleton and motility features as it is the case of Fibronectin, SNAI1, Ezrin and FHOD1. With the use of puromycin labelling we have co-localized active ribosomes with eIF-5A2 not only in cytosol but also in areas of cellular protrusion. We have shown the bulk invasive capacity of cells overexpressing eIF-5A2 in mice.

**Conclusions:**

We propose the existence of a coordinated temporal and positional interaction between TFGB and eIF-5A2 pathways to promote cell migration in NSCLC. We suggest that the co-localization of actively translating ribosomes with hypusinated eIF-5A2 facilitates the translation of key proteins not only in the cytosol but also in areas of cellular protrusion.

Video Abstract

**Supplementary Information:**

The online version contains supplementary material available at 10.1186/s12964-023-01076-6.

## Background

Translational control is essential to maintain cell function and is a crucial component in cancer development and progression. Alterations in the translation machinery lead to changes in protein synthesis and translation of specific mRNAs that promote tumor growth and metastasis [1].

The non-canonical eukaryotic translation initiation factor 5A (eIF-5A) is a small, essential and abundant protein that is highly conserved throughout evolution [2]. Human eIF-5A consists of two different isoforms encoded by *EIF5A1* and *EIF5A2* genes located in chromosome 17 [3] and 3 [4] respectively. The coding sequence of *EIF5A1* and *EIF5A2* share 80% identity and their proteins are 84% identical and 94% similar [4]. However, while eIF-5A1 is abundant and ubiquitously expressed in most tissues and cells [5], eIF-5A2 is rarely expressed in most tissues except in brain and testis, and it is overexpressed in several human cancers [4–7].

eIF-5A1 and eIF-5A2 are the only known proteins that undergo a post-translational modification, unique in nature, that generates the hypusine residue, which is essential for its activity [8]. Hypusination occurs through a pathway that includes two consecutive enzymatic reactions carried out by the enzymes deoxyhypusine synthase (DHS) and deoxyhypusine hydroxylase (DOHH) to modify the conserved Lys^50^ residue of eIF-5A. The first enzymatic step catalyzed by DHS uses the aminobutyl moiety of spermidine as a donor to generate the deoxyhypusinated residue which is subsequently hydroxylated by DOHH to render the hypusinated eIF-5A, which is the active and functional form of the factor in the ribosome [7, 9]. Notably, both enzymatic steps of the hypusination modification are susceptible of drug inhibition [10–12].

The best studied eIF-5A protein is by far eIF-5A1. Its biological function, in addition to translation, has been related to mRNA turnover, and nucleocytoplasmic transport [7]. Its original description as translation initiation factor [13] was later reassigned to the step of translation elongation [14]. More specifically its role during translation elongation in the ribosome has been allocated to conditions of ribosome stalling caused by the presence of polyproline domains or other specific motifs that slow-down ribosomal advance [15–17]. It has been identified as a tumor suppressor in lymphoma [18]. In contrast, tumor promoting actions for eIF-5A1 have been described in glioblastoma [19], colorectal cancer [20], cervical [21] and epithelial ovarian cancer [22].

eIF-5A2 function has been related to the regulation of transcription but not protein synthesis [23, 24]. It is overexpressed in tumor tissues [7, 25–28], and its overexpression leads to metastasis in hepatocellular carcinoma [29], non-small cell lung cancer [30]**,** esophageal squamous cell carcinoma [31] and bladder cancer [32]. Since eIF-5A2 is not widely expressed in normal tissue and its overexpression is associated with cancer, it has been postulated as a marker of bad prognosis and more aggressive stage of the disease [30, 33, 34]. In addition, the fact that eIF-5A2 knock-out mouse were viable and had normal development [12] made eIF-5A2 a very attractive therapeutic target in cancer. However, the regulation of eIF-5A2, its modification by hypusination and its functional role as translation factor in cancer cells undergoing migration and invasion is still poorly understood.

In the present study we have addressed the role of eIF-5A2 in lung adenocarcinoma (LUAD), the most common histological type of non-small cell lung cancer (NSCLC). We have compared the different cellular phenotypes by altering eIF-5A1 or eIF-5A2 expression in cell proliferation and migration of NSCLC cell lines. Additionally, we have addressed the role of eIF-5A2-mediated translational control in TGFB1-activated EMT, and the results obtained has allowed us to propose a novel function for eIF-5A2 as a key regulator in the translation of proteins involved in the migration and invasion properties of NSCLC cells.

## Methods

### Cell culture and expression vectors, reagents and antibodies

A549 and H1395 cells were cultured in DMEM/F12 supplemented with 10% foetal bovine serum. H1395 stable cell lines were generated transfecting cells with pCIG-IRES-GFP (empty vector) and pCIG-Flag-eIF-5A2-IRES-GFP using Lipofectamine 3000 (Invitrogen). Transfected cells were selected by resistance to G418 antibiotic and GFP positive cell populations were sorted by flow cytometry (MoFlo XDP, Beckman-Coulter). TGFB1 ligand (Pepro-Tech) was added to a final concentration of 10 ng/mL. Puromycin was added at a final concentration of 50 µg/mL for 5 min at 37 °C. The antibodies used for western blotting are listed in Additional file 1: Table S1. Primary and secondary antibodies used for immunohistochemistry and immunofluorescence microscopy are listed in Additional file 1: Tables S2 and S3.

### NSCLC patient samples

Seven samples from NSCLC patients of normal tissue (NT) and primary tumor (PT) were included in this study (Additional file 1: Table S4) and [35]. Fixed samples from these biopsies were used for the construction of a Tissue microarray (TMA) including two cylinders per sample. The TMA was used for immunohistochemical analyses as described in [35].

Frequencies of *EIF5A1* and *EIF5A2* gene alterations and Z-score of *EIF5A1* and *EIF5A2* amplification in LUAD cancer dataset (TCGA Pan-Cancer Atlas Studies) were obtained from cBioPortal for Cancer Genomics [36] in November 2022.

### RNA interference experiments and RT-qPCR

Cells were transfected with 200 ng of *EIF5A1* and *EIF5A2* siRNA (Additional file 1: Table S5) or with negative control siRNA using Lipofectamine 3000 (Invitrogen). After 24 h, TGFB1 ligand was added and medium with TGFB1 was replaced every day. The gene silencing effect was measured by western blotting and qPCR 72 h post transfection and 48 h hours post treatment.

RT-qPCR were performed as described in [37]. The primers used for PCR are listed in Additional file 1: Table S5.

### Fluorescence microscopy

Fluorescence microscopy was performed as described in [37]. For actin cystoskeleton staining, cells were first incubated with 0.2 mg/ml of DSP (dithiobis(succinimidyl propionate) and washed with 0.1 M glycine DMEM and then with PBS. The samples were then incubated with TSB at pH 6.9 (1 mM EGTA, 4% polyethylene glycol 6000, 100 mM PIPES and 0.5% Triton x-100 in distilled water). Next, cells were fixed with 4% PFA and washed with 0.1 M glycine in PBS. Cells were permeabilized with 1% Triton X-100 in PBS for 10 min and blocked for 1 h with 5% BSA in PBS. The primary and secondary antibodies were incubated under the conditions detailed in Additional file 1: Table S2 and S3. Actin filaments were labelled with Phalloidin-TRITC (Merck). The coverslips were mounted with Dako Ultramount permanent aqueous medium with DAPI (Agilent Dako). and analysed using a Leica TSC SP2 inverted confocal microscope (Leica Biosystems), a Leica TSC SP8 high-resolution multiphoton confocal microscope (Leica Biosystems), or an Apotome light microscope (Carl Zeiss).

### Polysome profiling

Cells were seeded in 150 mm plates and lysed as described by [38]. 10–50% sucrose linear gradients were performed on the Gradient Master (BioComp Instruments, Canada). The samples were ultracentrifuged at 230,000 × g for 2 h at 4 °C. Samples were fractionated and profiled with with the Piston Gradient Fractionator and the Triax^Tm^ flow cells software (BioComp Instruments, Canada). Total protein from the collected fractions was precipitated with trichloroacetic acid and used for western blot analysis.

### Cell proliferation assay (MTS)

Cells were seeded at a density of 1000 cells per well in a 96-well plate. Proliferation was evaluated 72 h after seeding with the kit CellTiter 96 Aqueous non-radiacitive Cell Proliferation Assay (Promega Corporation) according to standard protocols and analysed with a Victor 2 plate reader (Perkin Elmer). Cell titration assay was performed to optimize the initial cell number plated and to ensure linearity between cell density and MTS absorbance at 490 nm (Additional file 1: Fig. S3), according to the manufacturer instructions.

### Cell migration assay

Cells were seeded at 25–30% confluence in 96 well plate and treated as indicated. A wound on the monolayer was performed automatically with the AutoScratch (BioTek). Phase contrast images were taken every hour for 48 h in a BioSpa 8 system (BioTek) coupled with a Cyation 5 system (BioTek). Cell migration was analyzed with Gene 5 software (BioTek), to calculate the width of the wound over time. The width of the wound was analyzed by calculating the microns that the cells had traveled in each image taken with respect to the image taken at time 0.

### Xenograft mouse models

8 NSG mice (JAX^®^ Mice Strain, NOD.Cg-Prkdcscid Il2rgtm1WjI/SzJ, Charles River Laboratories) aged 5–6 weeks were included in the study without distinction by sex. Mice were injected subcutaneously with 500 H1395-EV and H1395-eIF-5A2 cells suspended in 200 µL of serum-free medium and Matrigel in a 1:1 ratio (*n* = 4/cell line). Mice behavior and tumor growth were observed over subsequent weeks and tumor growth was measured as described in [35]. Mice were euthanized with CO_2_ according to animal welfare criteria. Tumor, lungs, and liver from mice were fixed by immersion in 4% PFA. 7 µm thick lung sections were mounted on slides with Fluoromount-G aqueous mounting medium (Thermo Fisher Scientific). Images of random areas in each tissue section were taken in an Apotome light microscope.

### Statistical analysis

ANOVA and t-test analyses were performed using the GraphPad Prism software version 7.00 for Windows.

## Results

### eIF-5A2 is highly expressed in lung adenocarcinoma tumors and is associated with poor prognosis

**Fig. 1:**
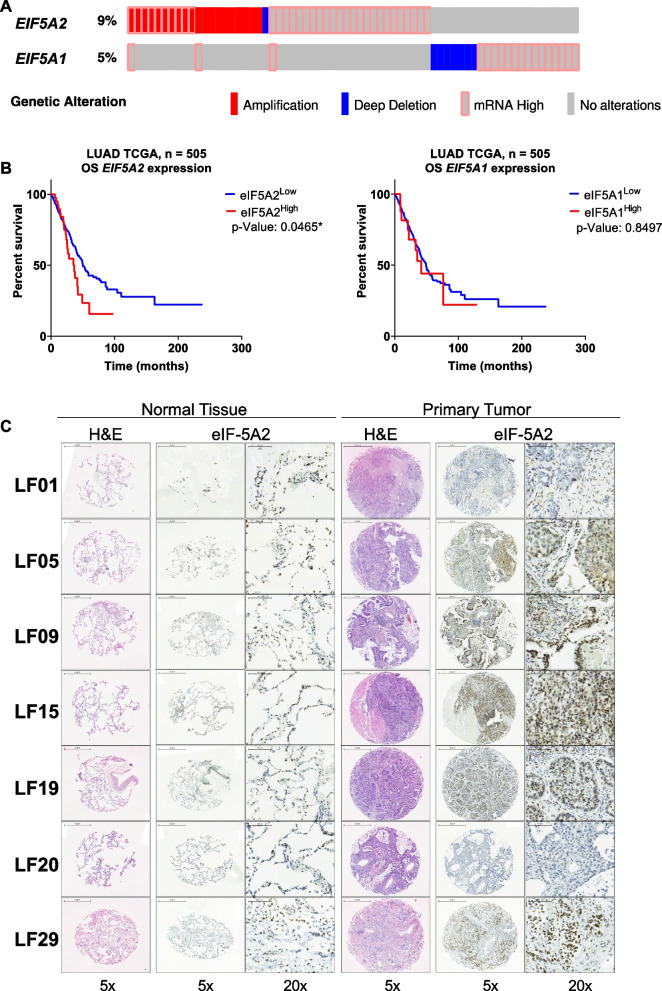
*EIF5A2* overexpression in LUAD is associated with poor survival. **A** Genomic alterations of *EIF5A1* and *EIF5A2* analysis using the dataset of TCGA Pan-Cancer Atlas Studies at the cBioPortal for Cancer Genomics. Green, mutation; red, amplification; blue, deep deletion. **B** Survival prognostic curve in patients with LUAD is shown. **C** eIF-5A2 localises in the nucleus, perinucleus and cytoplasm of cancer cells. In normal lung tissues eIF-5A2 is expressed in alveolar walls and cytoplasm of alveolar macrophages. Tissue microarray immunohistochemistry of the samples from patients LF01, LF05, LF09, LF15, LF19, LF20, LF21 and LF29 [35]. Normal lung tissue adyacent to primary tumor and primary tumor tissue are shown. Images show Hematoxylin and eosin (H&E) staining along immunohistochemistry with the anti-eIF-5A2 antibody. Sale bar for 5X images are 500 µm; scale bar for 20X images are 100 µm

We have scored the presence of genetic alterations of *EIF5A1 and EIF5A2* in LUAD data sets from TCGA with cBioportal for Cancer genomics [36]. We found that 9% of the tumor samples displayed amplification and high mRNA levels of *EIF5A2*, while 5% showed high levels of mRNA and deep deletion for *EIF5A1* gene (Fig. 1A). We next addressed whether high eIF-5A levels were associated with bad prognosis in LUAD and found that cancer patients with high *EIF5A2* levels showed poorer prognosis than the rest of patients (Fig. 1B). In contrast, high levels of *EIF5A1* mRNA were not associated with bad prognosis (Fig. 1B).

Next, we performed immunohistological analysis to evaluate the levels of eIF-5A2 protein and its localization in biopsy tumor tissue samples and normal adjacent tissue from 7 patients with early stage LUAD. eIF-5A2 was expressed in the cytoplasm, nucleus and perinucleus of carcinoma cells (Fig. 1C), while in adjacent normal lung samples eIF-5A2 immunological signal was strongly reduced and limited to the cytoplasm of alveolar walls and alveolar macrophages.

Altogether the genomic data from the Cancer Genomics Portal, together with individual analysis from 7 LUAD patients suggest that eIF-5A2 enhanced expression shows strong correlation with bad prognosis.

### Functional divergences between eIF-5A1 and eIF-5A2 on cell proliferation, cell migration and cytoskeleton organization

To discriminate between the roles of eIF-5A1 and eIF-5A2 in cell proliferation and cell migration in LUAD cells, we investigated the consequence of their genetic inhibition in A549 and H1395 cell lines. Since both *EIF5A1* and *EIF5A2* display high sequence similarity, siRNA sequences (si*EIF5A1* and si*EIF5A2*) were designed to specifically silence each gene (Additional file 1: Fig. S1).

**Fig. 2:**
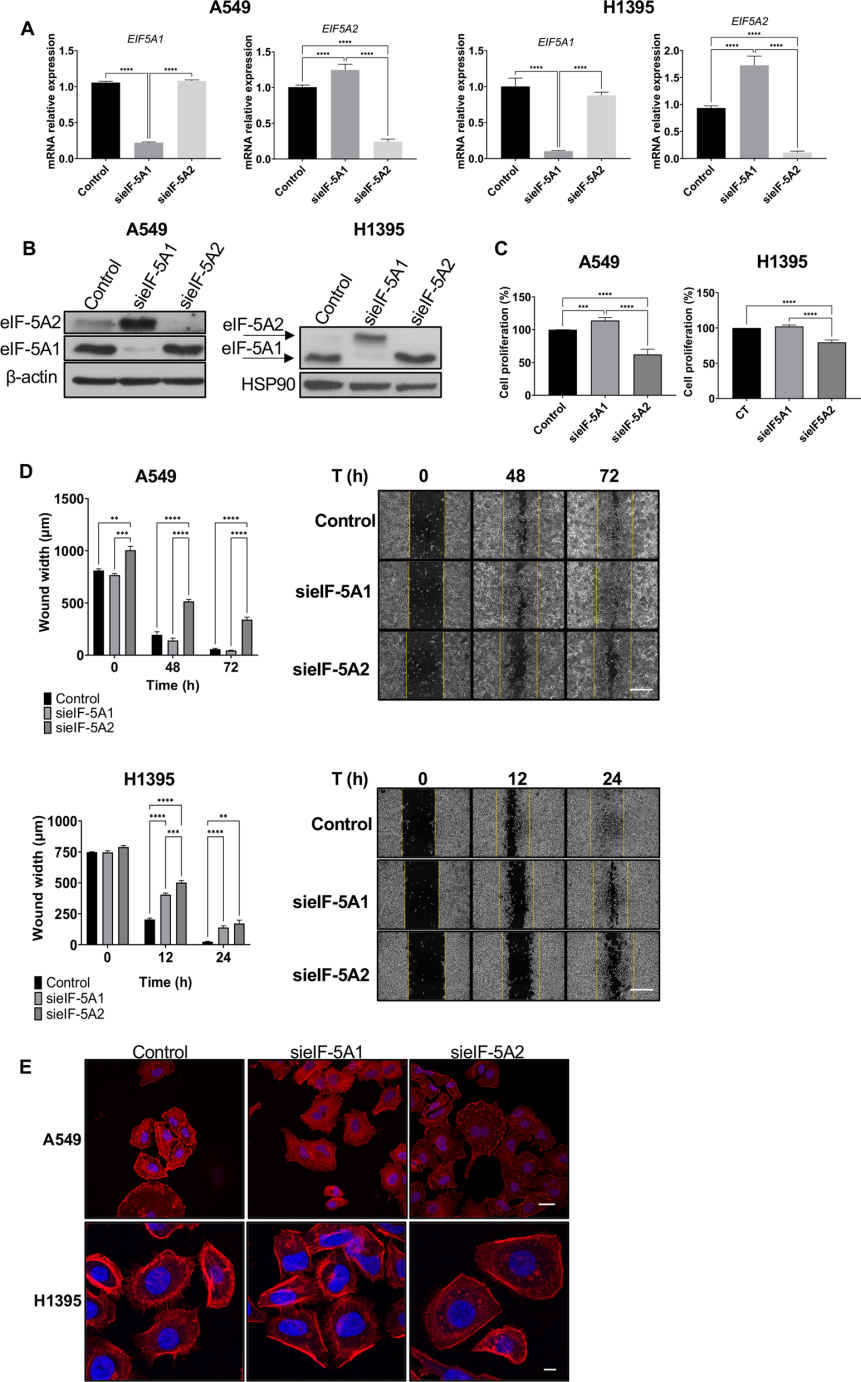
Differential contribution of eIF-5A1 and eIF5-A2 to cell proliferation and migration. **A** A549 and H1395 cells were transfected with either a control siRNA (Control) or *EIF5A1*- and *EIF5A2*-specific siRNAs (*siEIF5A1* and *siEIF5A2*) for 72 h. The mRNA expression was analyzed by RT-qPCR. Experimental means (*n* = 3 with experimental triplicates) were compared by two-way ANOVA analysis with Tukey's test for multiple comparison of samples (***p < 0.001, ****p < 0.0001). **B** eIF-5A1 and eIF-5A2 protein levels upon transfection of siRNA. eIF-5A1 and eIF-5A2 proteins levels were assayed by immunoblotting in A549 and H1395 cells processed as in (A). b-Actin or HSP90 were used as a loading control. A representative image of the experiments performed is shown (*N* = 3). **C** Cell *proliferation* was assayed using the MTS assay upon *siEIF5A1* and *siEIF5A2* transfection. Experimental means (*n* = 6 with experimental triplicates) were compared by two-way ANOVA with Tukey's test for multiple sample comparison (***p < 0.001, ****p < 0.0001).) **D** Analysis of cell migration in siRNA *EIF5A1* or siRNA *EIF5A2* transfected cells. Wound closure was performed 96 h after trasnfection and cell migration was monitored by phase-contrast microscopy. The means of the experiments (*n* = 3 with experimental triplicates) were compared by two-way ANOVA analysis with Tukey's test for multiple comparison of samples (**p < 0.01, ***p < 0.001, ****p < 0.0001) (left panel). Phase constrast representatives images. Scale bar 500 µm. **E** Genetic inhibition of *EIF5A1* and *EIF5A2* alters the organization of the F-actin citoeskeleton. Representative fluorescence microscopy images of A549 and H1395 cells transfected with si*EIF5A1* and si*EIF5A2* for 72 h. F-actin cytoskeleton was stained with rhodamine phalloidin (red). Dapi staining (blue) was used to visualised the nuclei. Scale bar of A549 fluorescence images 25 µm; scale bar of H1395 fluorescence images 10 µm

We confirmed the siRNA specificity by analysing the mRNA and protein expression of eIF-5A1 and eIF-5A2 by RTqPCR (Fig. 2A) and western blot (Fig. 2B) respectively. It should be remarked here that the western blot studies were performed with a different anti-eIF-5A2 antibody which also recognized, to a lower extent, the eIF-5A1 protein. Notably, we observed an increase in the levels of eIF-5A2 mRNA and protein in the cells transfected with sieIF-5A1 (Fig. 2A and B), but not the other way around, suggesting a unidirectional compensation between both homologs.

Next, cell proliferation was scored by MTS proliferation assay in the same cells with the same silencing constructs. The results showed that depletion of *EIF5A1* increased 14% and 2% cell proliferation of A549 and H1395 cells respectively (Fig. 2C). In contrast, depletion of eIF-5A2 decreased cell proliferation by 38% in A549 cells and 20% in H1395 cell line. In addition, we also tested cell migration alterations by wound healing assays (Fig. 2D). The results showed a decrease in cellular migration when depleting any of the genes, with a more drastic effect of eIF-5A2 depleted cells in comparison with eIF-5A1 depletion in both A549 and H1395 cell lines.

It has been shown that among the different actin polymerization proteins, formins are bona fide eIF5A client proteins as they contain large stretches of consecutive polyproline domains (Additional file 1: Fig. S2) [39]. To evaluate possible cytoskeleton alterations upon eIF-5A silencing, we stained A549 and H1395 cells transfected with si*EIF5A1*, si*EIF5A2* or siControl cells treated with rhodamine-phalloidin to visualize the structure of the stress actin cables. As shown in Fig. 2E, the depletion of eIF-5A2 altered the organization of the actin cytoskeleton of both A549 and H1395 cell lines to a larger extent than with si*EIF5A1* treatment. We could observe upon si*EIF5A2* transfection drastic effects like broken and disorganized actin fibers and cytoplasmic aggregates.

All together, these data suggest that eIF-5A1 and eIF-5A2 have different impact on cancer cell proliferation, migration and cytoskeleton rearrangements. While opposite effects were detected in cell proliferation, both genes seemed to behave similarly in cell migration assays and cytoskeleton alterations, although with a different degree, being more extreme for eIF-5A2 depletion.

### Overexpression of eIF-5A2 promotes cell proliferation and cell migration and is associated to polysomes

**Fig. 3:**
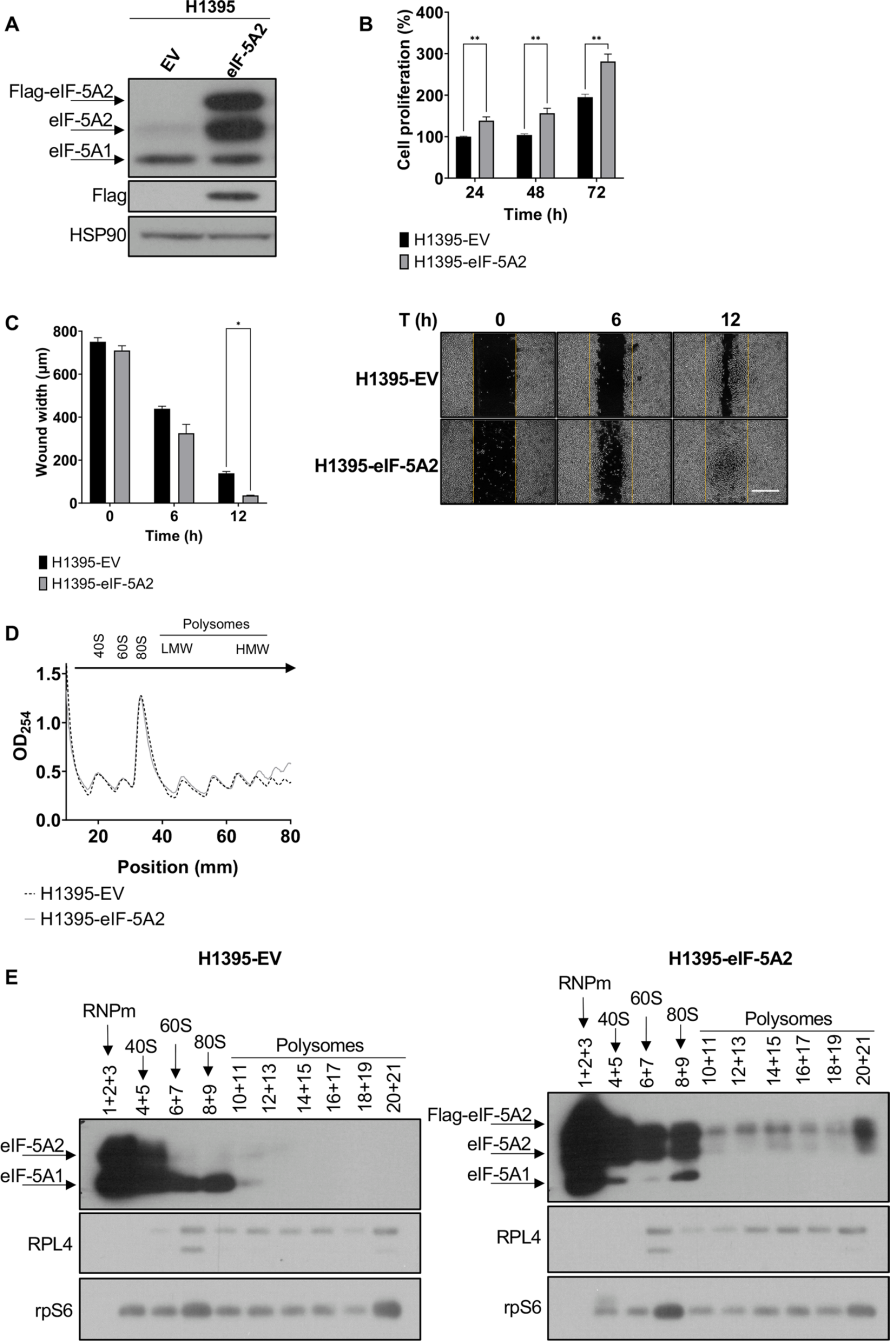
Overexpression of eIF-5A2 induces cell proliferation and migration, and enhances translation.** A** Protein extracts from empty vector (H1395-EV) or Flag-eIF-5A2 (H1395-eIF-5A2) stably-transfected H1395 cells were analysed by western blot with eIF-5A2 and Flag antibodies. HSP90 was used as a loading control protein expression. A representative image of the three idependent experiments is shown.** B** Cell proliferation assay in H1395-EV and H1395-eIF-5A2 cells. MTS assays were performed 24 h, 48 h and 72 h after seeding the cells. Experimental means (*n* = 6 with experimental triplicates) were compared by two-way ANOVA with Tukey's test for multiple sample comparison (**p < 0.01). **C** Cell migration assay in H1395-EV and H1395-eIF-5A2 cells. Wound was performed in confluent monolayer cell culture, and wound closure was monitored by phase-contrast microscopy. The means of the experiments (*n* = 3 with experimental triplicates) were compared by two-way ANOVA analysis with Tukey's test for multiple comparison of samples (*p < 0.05) (right panel). Representative images of phase contrast microscopy are shown in the left panel. Scale bar 500 µm. **D** Polysomal profile of H1395-EV and H1395-eIF-5A2 cells. Global RNA polysome profiles generated by measurement of the OD at 254 nm in the density gradient fractionation system are shown. From left to right, each peak represents the 40S subunit, the 60S subunit, monosomes (80S), disomes, and polysomes. A representative graph of the experiments performed is shown (*n* = 3). **E** Protein extracts from the fractions obtained in D were analysed by western blot with eIF-5A2, RPL4 and rpS6 antibodies. A representative image of the experiments performed is shown (*n* = 2)

To assess the effect in cell proliferation and migration upon eIF-5A2 overexpression, we generated an H1395 cell line stably expressing Flag-eIF-5A2 under the control of the pCIG-IRES-GFP promoter (H1395-eIF-5A2). As a control, we generated transgenic H1395 cells stably expressing pCIG-IRES-GFP empty vector (H1395-EV). We observed that endogenous eIF-5A1 protein expression was not altered in H1395-eIF-5A2 cells compared to H1395-EV cells (Fig. 3A). Cell proliferation assays by MTS showed that overexpression of eIF-5A2 increased cell proliferation by more than 85% compared with empty vector cells (Fig. 3B). In addition cell migration was also increased in cells overexpressing eIF-5A2 (Fig. 3C). These results suggest that overexpression of eIF-5A2 promotes both, cell proliferation and cell migration.

Next, we tested the association of eIF-5A2 protein isoforms to polysome fractions. Polysome profiling analysis in H1395-EV and H1395-eIF-5A2 showed an increase in the number of heavy polysomes in eIF-5A2 overexpressing cells, which suggests enhanced protein translation in these cells (Fig. 3D). In addition, we observed enhanced association of Flag-eIF-5A2 with polysomes (Fig. 3E, fractions 16–21) suggesting that overexpression of eIF-5A2 may be involved in the increased translational rate of these cells. Immunoblots of ribosomal proteins rpL4 and rpS6 were performed with the same fractions showing similar expression patterns (Fig. 3E).

The increase in eIF-5A2 association to translating ribosomes suggests that eIF-5A2 is involved in the synthesis of proteins possibly involved in cell proliferation and migration.

### TGFB1 induces eIF-5A2 expression

**Fig. 4:**
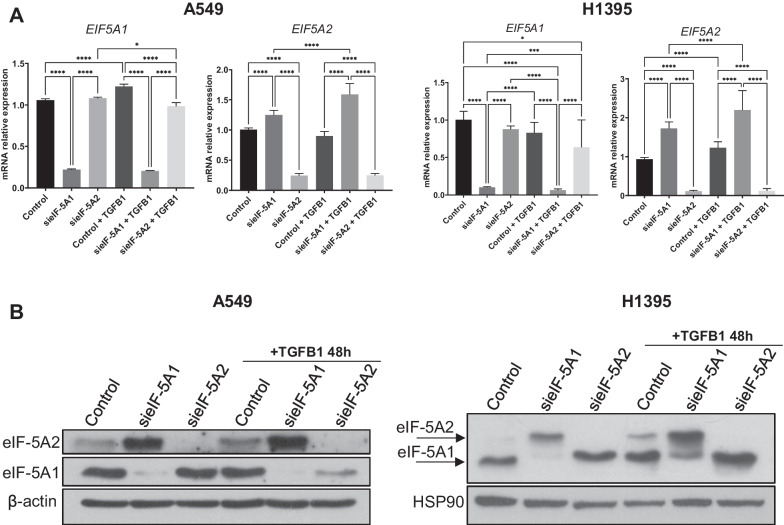
TGFB1 induces eIF-5A2 protein expression. **A** *EIF5A1* and *EIF5A2* mRNA levels in A549 and H1395 cells treated with TGFB1. Cells were transfected with si*EIF5A1*, si*EIF5A2* or siCT for 72 h and treated or not with TGFβ1 for 48 h. The mRNA relative expression was analyzed by RT-qPCR. Experimental means (*n* = 3 with experimental triplicates) were compared by two-way ANOVA analysis with Tukey's test for multiple comparison of samples (*p < 0.05, **p < 0.01, ****p < 0.0001). **B** eIF-5A2 and eIF-5A1 protein levels of cells in A were analised by western blot. β-Actin or HSP90 were used as a loading control. A representative image of the experiments performed is shown (*n* = 3)

eIF-5A2 has been previously involved in the epithelial to mesenchymal transition (EMT) in LUAD [40]. One of the signaling pathways involved in EMT activation is transforming growth factor beta (TGF-β) [41–43]. We addressed whether TFGB1-induced EMT signaling affects eIF-5A1 and eIF-5A2 expression in A549 and H1395 cells. First, we analyzed the mRNA levels of *EIF5A1* and *EIF5A2* in cells treated or not with TGFB1 peptide. We observed that addition of TGFB1 induced subtle changes in the levels of *EIF5A1* and *EIF5A2* mRNA (Fig. 4A), however, when silencing *EIF5A1,* TGFB1 treatment provoked a notable increase in the expression of *EIF5A2*.

We then analysed the protein levels and observed a drastic positive effect of TFGB1 treatment on the expression of eIF-5A2 protein compared to the non-treated control cells. Similar to the transcriptional data, no obvious effect of TFGB1 could be detected on eIF-5A1 level (Fig. 4B).

We can conclude that the TFGB1-dependent EMT activation leads to enhanced expression of eIF-5A2 with no consequence on eIF-5A1 protein.

### TGFB1 treatment induces hypusination of eIF-5A2 and eIF-5A2-dependent EMT protein expression

**Fig. 5:**
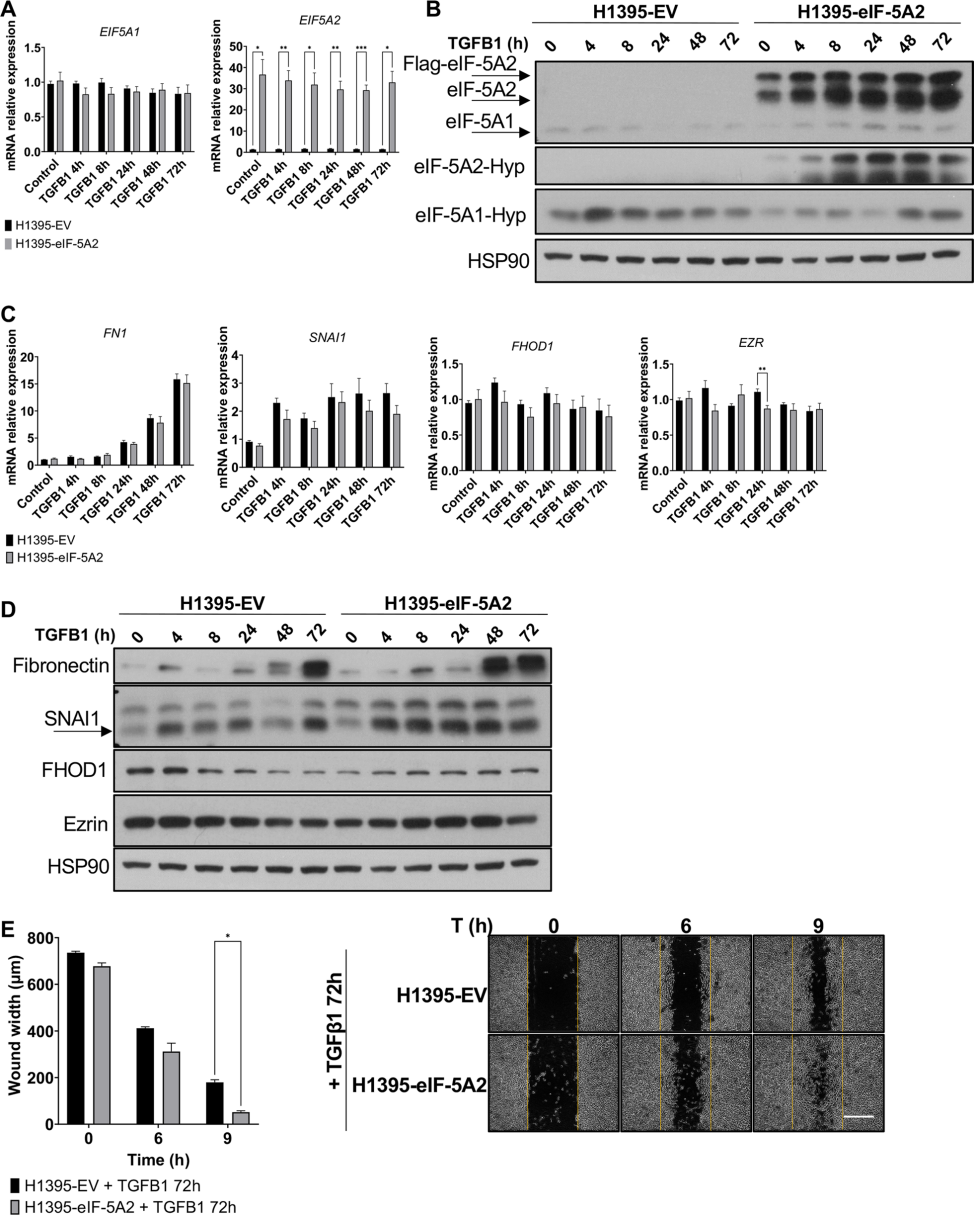
TGFB1 induces eIF-5A2 hypusination and eIF-5A2-dependent expression of EMT proteins. **A** *EIF5A1* and *EIF5A2* mRNA levels in H1395-EV and H1395-eIF-5A2 cells treated with TGFB1 for the indicated times. The mRNA expression was analyzed by RT-qPCR. Experimental means (*n* = 3 with experimental triplicates) were compared by two-way ANOVA analysis with Tukey's test for multiple comparison of samples (*p < 0.05, **p < 0.01, ***p < 0.001). **B** eIF-5A1 and eIF-5A2 protein levels and their hypsuinated forms of cells in A were analysed by western blot with eIF-5A2 and hypusine antibodies. HSP90 was used as a loading control. A representative image of the experiments performed is shown (*n* = 3). **C** mRNA levels of *FN1*, *FHOD1*, *EZRIN* and *SNAI1* of cells in A. The mRNA expression levels were analyzed by RT-qPCR. Experimental means (*n* = 3 with experimental triplicates) were compared by two-way ANOVA analysis with Tukey's test for multiple comparison of samples (*p < 0.05, **p < 0.01). **D** Protein expression of Fibronectin, FHOD1, Ezrin and SNAI1 of cells in A were analysed by western blot with the indicated antibodies. A representative image of the experiments performed is shown (*n* = 3). **E** Cell migration assays in H1395-EV and H1395-eIF-5A2 cells, in the presence of TGFB1 ligand for 72 h. Wound was performed in confluent monolayer cell culture, and wound closure was monitored by phase-contrast microscopy. The means of the experiments (*n* = 3 with experimental triplicates) were compared by two-way ANOVA analysis with Tukey's test for multiple comparison of samples (*p < 0.05) (left panel). Representative phase-contrast microscopy images are shown in the right panel. Scale bar 500 µm

To determine whether TGFB1-dependent induction of eIF-5A2 expression is transferred into higher eIF-5A2 activity, we analised the kinetics of the post-translational activation of eIF-5A2 by hypusination by means of western blot analsysis with H1395-eIF-5A2 and H1395-EV cell lines treated with TGFB1 for 72 h. As shown in Fig. 5A, neither *EIF5A1* nor *EIF5A2* mRNA levels were affected by TGFB1 treatment, although the *EIF5A2* mRNA levels were high due to the overexpression of *Flag-EIF5A2*. However, differences between eIF-5A1 and eIF-5A2 hypusination patterns were detected after TGFB1 treatment. Although the hypusination profile of eIF-5A1 did not display any alteration, the eIF-5A2 hypusination pattern was enhanced by the TGFB1 treatment showing a peak around 24–48 h (Fig. 5B).

We then investigated the role of eIF-5A2 in the acquisition of a mesenchymal phenotype induced by TGFB1. For this purpose, we analysed the expression of two mesenchymal markers, Fibronectin and SNAI1, which, as polyproline containing proteins (Additional file 1: Fig. S2) qualify as eIF-5A clients. In the cells treated with TGFB1 for 72 h, we observed an increase in the *FN1* mRNA levels (Fig. 5C), concomitant with an increase in its protein expression over time of TGFB1 exposure (Fig. 5D). Moreover, the expression of Fibronectin protein was higher in H1395-eIF-5A2 cells treated for 48 and 72 h in comparison to control cells. The expression kinetics of *SNAI1* mRNA during TGFB1 treatment showed a 2 to threefold increase in both cells lines (Fig. 5C). The protein levels of SNAI1 were higher in H1395-eIF-5A2 compared to control cells (Fig. 5D). Of note, the protein showed a transient increase during 4 to 48 h of TGFB treatment in H1395-eIF-5A2 cells suggesting a post-transcriptional regulation. These results confirmed that overexpression of eIF-5A2 promoted the expression of proteins inolved in the adquisition of a TGFB1-dependent mesenchymal phenotype.

Other polyproline containing proteins involved in the EMT differentiation are Ezrin (Additional file 1: Fig. S2), that connects the actin cytoskeleton with the cell membrane [44] and the actin polymerization formin FHOD1 [45]. The expression of *FHOD1* and *EZR* mRNA were similar in both cell lines and had minor changes during the TGFB1 treatment (Fig. 5C). We also analised the expression of FHOD1 and Ezrin proteins in H1395-EV and H1395-eIF-5A2 cells treated with TGFB1 (Fig. 5D). The protein levels of FHOD1 decreased after TGFB1 treatment in control H1395-EV cells. In contrast, the expression of FHOD1 increased during TGFB1 treatment in H1395-eIF-5A2 cells. Similarly, Ezrin protein levels continuously decreased in H1395-EV cells, while a sustained high protein level was observed during the period of TGFB1 treatment in H1395-eIF-5A2 until 72 h when protein levels dropped (Fig. 5D). These results suggest that eIF-5A2 overexpression did promote up-regulation of FHOD1 and Ezrin at the post-transcriptional level.

Finally, we analysed cell migration by wound healing assays of these cells upon TGFB1 treatment. The results, displayed in Fig. 5E, showed an increase in H1395-eIF-5A2 migrating cells, which were capable of closing the wound after 12 h, unlike H1395-EV cells in which the wound stayed unclosed. These results are in agreement with data shown in Fig. 2D where inhibition of eIF-5A2 expression repressed cell migration.

### eIF-5A2 colocalizes with active translation sites and promotes bulk cell invasion

**Fig. 6:**
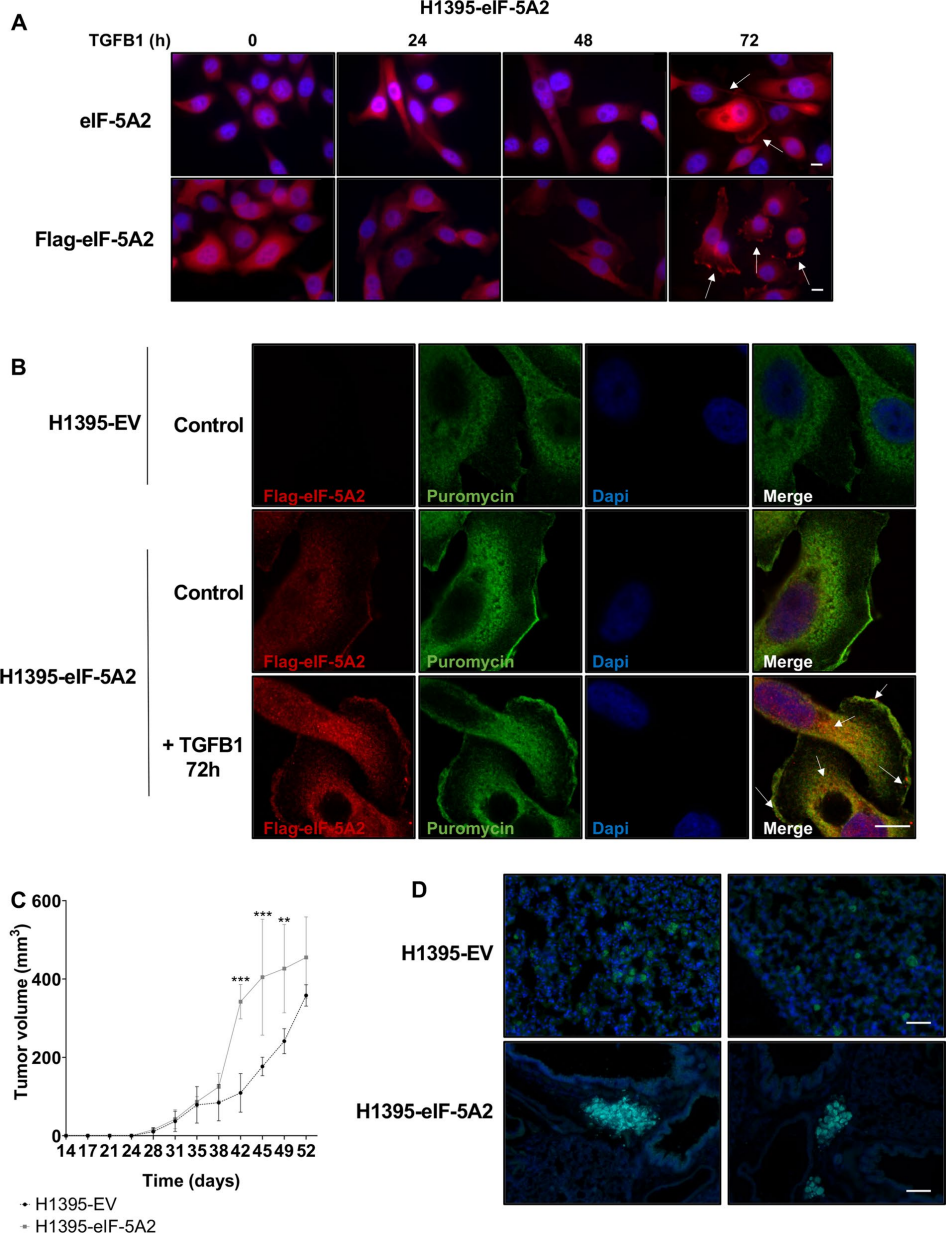
Overexpression of eIF-5A2 colocalizes with puromycin and promotes tumor metastasis in vivo. **A** Representative fluorescence microscopy images of H1395-eIF-5A2 cells untreated or treated with TGFB1 for the indicated times. Cells were stained with anti-eIF-5A2 or anti-Flag antibodies, and Dapi (blue) to observe the nuclei. Scale bar 10 µm. **B** Visualisation of the translational activity of eIF-5A2. H1395-EV and and H1395-eIF-5A2 treated with puromycin in the presence or absence of TGFB ligand for 72 h were stained with anti-Flag (red) and anti-Puromycin (green) antibody to visualize active places of translation in cells. Dapi (blue) staining was used to visualize nuclei. Scale bar 10 µm. **C** Overexpression of eIF-5A2 promotes tumor metastasis in vivo. H1395-EV and H1395-eIF-5A2 cells were subcutaneously injected in NSG mice. Tumor volume in the subcutaneous xenograft model was measured every 3–4 days while the condition of the mice was checked. Approximately two months after implantation, mice were euthanized and dissected (left panel). **D** Representative fluorescence microscopy images of lung metastasis of NSG mice injected as described in C. H1395-EV and H1395-eIF-5A2 metastasic cells expressing GFP is shown in green. Tissue was stained with Dapi (blue) to visualize nuclei. Scale bar for H1395-EV cells images 50 µm; scale bar for H1395-eIF-5A2 cells images 100 µm)

TGFB1 treatment induces changes in the cytoskeleton to allow the cell to adquire a mesenchymal phenotype. Thus, we wanted to analyse whether the subcellular localisation of eIF-5A2 could be altered upon TGFB1 treatment. In fact, we observed that in TGFB1-treated cells for 72 h eIF-5A2 acumulates in the cell protrusions that appeared after 72 h of TGFB1 treatment (Fig. 6A).

We also analysed the subcellular localization of actively translating eIF-5A2. To this aim we perfomed colocalization of eIF-5A2 and puromycin in H1395-eIF-5A2 cells treated with TGFB1. Puromycin is a tyrosyl-tRNA mimic that blocks translation by labeling and releasing elongating polypeptide chains from translating ribosomes [46], therefore antibodies against puromycin were used to localize translating ribosomes in the cells [47].

We observed that eIF-5A2 colocalized with puromycin close to the cell membrane and in filopodia (Fig. 6B), suggesting that eIF-5A2 may be involved in the translation of specific proteins in the same places where they are required to activate morphological changes.

We also used anti-Flag antibodies to specifically visualize the exogenous eIF-5A2 protein and observed that, in the H1395- eIF-5A2 cells treated with TGFB1, the levels of immunolabelled puromycin increased (Fig. 6B). These data indicate a higher translational rate of H1395-eIF-5A2 cells compared to H1395-EV cells, in agreement with the results shown in Fig. 3D. In addition, positional colocalization of Flag-eIF-5A2 with puromycin confirmed active translation places where eIF-5A2 may be required to actively translate polyproline proteins involved in morphological rearrangements (Fig. 6B).

Finally, the invasive capacity of H1395-EV and H1395-eIF-5A2 cells was studied in an in vivo model. To this aim, 500 cells of each cell line were injected into immunocompromised mice subcutaneously. Although there were no differences in the final tumor volume generated by the two cell lines, we observed that the cells overexpressing eIF-5A2 increased tumor volume faster that H1395-EV cells, which had a more linear growth (Fig. 6B). As these tumors showed differential growth, we analised whether cancer cells had been able to migrate and colonize other tissues once the tumor had reached its maximum size. This analysis was facilitated by the GFP-labelling of xenografted cells, as the plasmid used to generate them is a bicistronic vector expressing GFP protein from an IRES sequence. We performed immunofluorescence on sections of lung tissue collected at the time of euthanasia, and we were able to confirm a difference in the invasive capacity of the cells. H1395-EV cells that reached the lung tissue invaded it as individual cells, while H1395-eIF-5A2 cells were capable of forming larger bulks of cells in the lungs (Fig. 6D).

## Discussion

Human translation factor eIF-5A2 isoform is frequently overexpressed in lung adenocarcinoma. It has been considered as a valuable and safe target for therapeutic intervention in cancer, since it is dispensable for normal development and viability, however its regulation and function is not completely understood. In the present study we have investigated the pathogenic role of eIF-5A2 in LUAD. We have shown that eIF-5A2 is amplified in 9% of LUAD patients and its overexpression is associated with poor prognosis. These data were correlated with our experimental evidence of a higher cell invasion capacity when cells overexpressing eIF-5A2 were grafted into immunocompromised mice. We have also analysed the effects of genetic silencing of eIF-5A1 and eIF-5A2 isoforms in LUAD cells to study their contribution to cancer progression. By studying the phenotypic consequences of the downregulation of both genes we observed that, while eIF-5A2 function preferentially regulated cell migration, eIF-5A1 function was more related to cell proliferation. Additionally, overexpression of eIF-5A2 led to both enhanced proliferation and cell migration in LUAD. A functional link to the TGFB1 pathway was also uncovered showing that TGFB treatment led to enhanced expression of eIF-5A2 protein as well as a transient expression of hypusinated eIF-5A2, thus boosting the translation of polyproline containing proteins involved in the cellular migration process.

One important aspect of our investigations, which is often underestimated, is the genetic compensation between the eIF-5A isoforms. We have shown that depletion of eIF-5A1 induced upregulation of eIF-5A2 expression, but it did not occur in the opposite way. Thus, targeted elimination of *EIF5A1* should be taken wih care as a side effect leading to *EIF5A2* overproduction could have colateral consequences.

Depletion of both isoforms led to desorganization of the actin cytoskeleton, being this alteration more dramatic in eIF-5A2 depleted cells. This is important since the development of an invasive phenotype characteristic of cancer progression requires loss of cell polarity, cytoskeleton reorganization and cell shape reprogramming to increase the motility of individual cells. These are basic hallmarks of the epithelial-mesenchymal transition. How do cells orchestrate these morphological changes is a subject of extensive research, however there are still questions to be solved. The recent demonstration that eIF-5A translation factor is required for the translation of proteins with consecutive Pro residues opens new avenues of exploring the existence of eIF-5A translation hubs during EMT. Reinforcing this idea, gene ontology studies showed an enrichment of polyproline-rich proteins involved in cytoskeleton organization like the formins, with direct functions in cell morphology, adhesion and migration [48]. This idea was later confirmed with functional studies [39, 49]. However, unlike eIF-5A1, eIF-5A2 has been implicated in the regulation of transcription but not in protein translation [23]. Here we show that eIF-5A2 overexpression is associated to translational regulation in LUAD.

To study the role of eIF-5A2 in LUAD cells in a microenvironment that facilitates EMT, we exogenously applied TGFB1 causing an enhanced expression of eIF-5A2 protein as well as a transient expression of hypusinated eIF-5A2. Importantly, the increased expression of hypusinated eIF-5A2 was associated with induction of SNAI1, Fibronectin, FHOD1 and Ezrin at the protein level. All these are polyproline containing proteins involved in the EMT, cell migration and invasion, and metastasis. Intriguingly, TGFB1 treatment induced localization of eIF-5A2 at the edge and protrusions of the cells undergoing EMT, places associated with traslational activity.

## Conclusions

In summary, our data showed that amplification of eIF-5A2 associates with poor outcome in LUAD, and reveal a novel potential role for eIF-5A2 to conform a specific translation hub for polyproline-rich proteins whose coordinated translation in predetermined subcellular locations is crucial for cell migration and invasion. Further studies should focus in the understanding of how are these specialized ribosomal hubs organized and guided to accomplish their functional role in LUAD metastasis to uncover potential anticancer targets.

## Supplementary Information

**Additional file 1. Figure S1.** Design of specific oligonucleotides for siRNA of EIF5A1 and EIF5A2. **Figure S2.** Fibronectin, FHOD1, Ezrin and SNAI1 protein sequence. **Figure S3.** Linearity between cell density and MTS absorbance at 490 nm. **Table S1.** List of primary antibodies used for western blot. **Table S2.** List of primary antibodies used for immunohistochemistry and immunofluorescence. **Table S3.** List of secondary antibodies used for immunofluorescence. **Table S4.** Summary of the clinicopathological and immunohistochemical features of the patients included in the study. **Table S5.** List of oligonucleotide sequences used in RT-qPCR.

## Data Availability

All data are available in the main text or the supplementary materials.
