# Supplementary material for: Eukaryotic Initiation Factor 5A2 localizes to actively translating ribosomes to promote cancer cell protrusions and invasive capacity
Source: Cell Commun Signal. 2023 Mar 13;21:54. doi: 10.1186/s12964-023-01076-6 (PMC10009989; doi:10.1186/s12964-023-01076-6)
Supplement: Supplementary file 2 — Additional file 1. Figure S1. Design of specific oligonucleotides for siRNA of EIF5A1 and EIF5A2. Figure S2. Fibronectin, FHOD1, Ezrin and SNAI1 protein sequence. Figure S3. Linearity between cell density and MTS absorbance at 490 nm. Table S1. List of primary antibodies used for western blot. Table S2. List of primary antibodies used for immunohistochemistry and immunofluorescence. Table S3. List of secondary antibodies used for immunofluorescence. Table S4. Summary of the clinicopathological and immunohistochemical features of the patients included in the study. Table S5. List of oligonucleotide sequences used in RT-qPCR. [file 12964_2023_1076_MOESM2_ESM.docx]

Supplementary Materials for

**Eukaryotic Initiation Factor 5A2 localizes to actively translating ribosomes to promote cancer cell protrusions and invasive capacity**

Arantxa Martínez-Férriz, Carolina Gandía, José Miguel Pardo-Sánchez, Alihamze Fathinajafabadi, Alejandro Ferrando and Rosa Farràs

Correspondence to: rfarras@cipf.es

**This file includes:**

Tables S1 to S5

Figs. S1 to S3

| **Antibody** | **Specie** | **Dilution** | **Reference** |
| --- | --- | --- | --- |
| **Anti-eIF-5A2** | **Rabbit polyclonal** | **1:1000** | **Proteintech Group, 17069-1-AP** |
| **Anti-Ezrin** | **Mouse monoclonal** | **1:1000** | **Thermo Fisher Scientific, MA5-13862** |
| **Anti-FHOD1** | **Rabbit polyclonal** | **1:500** | **Merck Millipore, ABS53** |
| **Anti-Flag** | **Mouse monoclonal** | **1:1000** | **Sigma-Aldrich Corporation, F3165** |
| **Anti-Fibronectin** | **Rabbit polyclonal** | **1:500** | **GeneTex, GTX112794** |
| **Anti-Hypusine** | **Rabbit polyclonal** | **1:5000** | **Merck Millipore, ABS1064** |
| **Anti-HSP90** | **Mouse monoclonal** | **1:5000** | **Santa Cruz Biotechnology, sc-13119** |
| **Anti-SNAI1** | **Rabbit polyclonal** | **1:500** | **GeneTex, GTX125918** |

**Table S1**. List of primary antibodies used for western blot.

| **Antibody** | **Specie** | **Dilution** | **Reference** |
| --- | --- | --- | --- |
| **Anti-eIF-5A2** | **Rabbit polyclonal** | **1:250** | **Sigma-Aldrich Corporation, HPA029090** |
| **Anti-Flag** | **Rabbit polyclonal** | **1:200** | **Cell Signaling, #2368T** |
| **Anti-Puromycin** | **Mouse monoclonal** | **1:1000** | **Merck Millipore, MABE343** |

**Table S2**. List of primary antibodies used for immunohistochemistry and immunofluorescence.

| **Antibody** | **Specie** | **Dilution** | **Reference** |
| --- | --- | --- | --- |
| **Anti-mouse IgG Texas red** | **Goat polyclonal** | **1:600** | **Invitrogen, T862** |
| **Anti-rabbit IgG Alexa fluor 633** | **Goat polyclonal** | **1:600** | **Invitrogen, A21072** |

**Table S3**. List of secondary antibodies used for immunofluorescence.

|  |  |  |  | **eIF-5A2** | | |
| --- | --- | --- | --- | --- | --- | --- |
| **Patient code** | **TNM Stage** | **Histology** | **Follow-up time (months)** | **Sample** | **Intensity** | **Localization** |
| **LF01** | **IIb** | **ADC (mainly acinar)** | **31,53** | **Normal tissue** | **+/-** | **Nucleus** |
|  |  |  |  | **Primary tumor** | **+/-** | **Nucleus** |
| **LF05** | **IVa** | **ADC (mainly solid)** | **29,23** | **Normal tissue** | **+/-** | **Nucleus** |
|  |  |  |  | **Primary tumor** | **+** | **Cytoplasm, perinuclear** |
| **LF09** | **Ib** | **ADC (mucinous mailny lepidic)** | **27,57** | **Normal tissue** | **+/-** | **Nucleus** |
|  |  |  |  | **Primary tumor** | **+/-** | **Cytoplasm, perinuclear, nucleus** |
| **LF15** | **IIb** | **ADC (mainly solid)** | **14,23** | **Normal tissue** | **+/-** | **Nucleus** |
|  |  |  |  | **Primary tumor** | **++** | **Cytoplasm, perinuclear, nucleus** |
| **LF19** | **Ia3** | **ADC (acinar)** | **24,30** | **Normal tissue** | **+/-** | **Nucleus** |
|  |  |  |  | **Primary tumor** | **++/-** | **Perinuclear, nucleus** |
| **LF20** | **IIIa** | **ADC (acinar)** | **12,10** | **Normal tissue** | **+/-** | **Nucleus** |
|  |  |  |  | **Primary tumor** | **+/--** | **Mainly perinuclear** |
| **LF29** | **IVb** | **ADC (solid poorly differentiated)** | **15,90** | **Normal tissue** | **+/-** | **Nucleus** |
|  |  |  |  | **Primary tumor** | **++/-** | **Perinuclear, nucleus** |

**Table S4**. Summary of the clinicopathological and immunohistochemical features of the patients included in the study. Samples from patients LF01, LF05, LF09, LF15, LF19, LF20, LF21 and LF29 were included in the study (n=7). ADC: adenocarcinoma.

| **Gene** | **Oligonucleotide sequence (5’-3’)** |
| --- | --- |
| **siRNA *EIF5A1* sense** | GUACGACUGUGGAGAAGAG |
| **siRNA *EIF5A2* sense** | GACAGAAACUGGUGAAGUU |
| ***EIF5A2*** | **Forward: AACTGCCAGAAGGTGAACTAGG** |
|  | **Reverse: GTTTCCGTTTATTTGCAGGGT** |
| ***EIF5A2* (plasmid)** | **Forward: GGCTTCCAGCACTTACCCTA** |
|  | **Reverse: ATGGTCGTCCTTTGAGCACC** |
| ***EIF5A1*** | **Forward: TTGGCAAGGAGATTGAGCAG** |
|  | **Reverse: TTTGCCATGGCCTTGATTGC** |
| ***EZR*** | **Forward: CTGAGACTGCCGTGCTCTTG** |
|  | **Reverse: GTAAGTTTGTGCTGGTCCATCACT** |
| ***FN1*** | **Forward: TGCTTCCTGGCCGAAAATAC** |
|  | **Reverse: ATCAGGCGCTGTTGTTTGTG** |
| ***FHOD1*** | **Forward: GCCAGCTGATGCTCTTTGTG** |
|  | **Reverse: CAGCTTCAGGGCTGTCTTCA** |
| ***MTA1*** | **Forward: AGATACGTGCAGCAGAAACG** |
|  | **Reverse: TTGACGCTGATTTGGTTCGG** |
| ***SNAI1*** | **Forward: CTAGGCCCTGGCTGCTACAA** |
|  | **Reverse:CCTGGCACTGGTACTTCTTGA** |
| ***S26*** | **Forward: CTGCACTAACTGTGCCCGATGCGTG** |
|  | **Reverse: GACGCTCGCTTCAGAAATGTCCCTG** |

**Table S5**. List of oligonucleotide sequences used in RT-qPCR.

**Figure S1**. *Design of specific oligonucleotides for siRNA of EIF5A1 and EIF5A2.*

**Figure S2**. *Fibronectin, FHOD1, Ezrin and SNAI1 protein sequence*. The consecutive proline motifs are marked in red.

**Figure S3**. *Linearity between cell density and MTS absorbance at 490 nm*. MTS cell proliferation assays in H1395-EV and H1395-eIF-5A2 cells were performed 72h after seeding the indicated number of cells per well. Simple linear regression of experimental means (N=3 with experimental triplicates) was analysed.
